# The weekend effect in pelvic fractures and influence of weekday and weekend accident days: a retrospective study of the German Pelvic Registry

**DOI:** 10.1038/s41598-025-98121-w

**Published:** 2025-04-29

**Authors:** Christof K. Audretsch, Maximilian M. Menger, Andreas Höch, Tina Histing, Mika F. Rollman, Benedikt J. Braun, Markus A. Küper, Steven C. Herath, Working Group on Pelvic Fractures of the German Trauma Society, Steven Herath, Andreas Höch, Alexander Hofmann, Alexander Trulson, Wolfgang Lehmann, Uwe Schweigkofler, Ulrich Stöckle, Ulf Culemann, Tim Pohlemann, Fabian Stuby, Markus Küper, Thomas Mendel, Thomas Fuchs, Sven Märdian, Daniel Wagner, Suzanne Zeidler, Silvan Wittenberg, Philipp Schwabe, Philipp Pieroh, Maximilian Hartel, Markus Beck, Mario Perl, Lisa Wenzel, Klaus-Dieter Schaser, Jan Friederichs, Hans-Georg Palm, Hagen Schmal, Friederike Klauke, Eftychios Bolierakis, Christopher Spering, Christian Zeckey, Georg Osterhoff, David Osche, Marcel Mäder, Ivan Marintschev

**Affiliations:** 1 Department for Traumatology and Reconstructive Surgery, BG Trauma Center, University of Tübingen, Tübingen, Germany; 2 Department of Orthopedics, Trauma and Plastic Surgery, University of Leipzig, Leipzig, Germany; 3 Department of Orthopedics and Traumatology, Westpfalz-Clinics, Hellmut-Hartert-Str. 1, 67655 Kaiserslautern, Germany; 4 Department of Trauma Surgery, BG Trauma Center, Murnau am Staffelsee, Germany; 5 Department of Trauma Surgery, Orthopaedics and Plastic Surgery, University of Göttingen, Robert-Koch-Str. 40, 37075 Göttingen, Germany; 6 Department of Trauma and Orthopedic Surgery, BG Unfallklinik Frankfurt, 60389 Frankfurt, Germany; 7 Center for Musculoskeletal Surgery, Charité-Universitätsmedizin Berlin, Corporate Member of Freie Universität Berlin and Humboldt-Universität zu Berlin, 10117 Berlin, Germany; 8 Clinic for Trauma Surgery, General Hospital Celle, Siemensplatz 4, 29221 Celle, Germany; 9 Department of Trauma, Hand, and Reconstructive Surgery, Saarland University, Homburg, Germany; 10 Department of Trauma and Reconstructive Surgery, BG Hospital Bergmannstrost Halle, Halle, Germany; 11 Center for Musculoskeletal Surgery, Vivantes Klinikum, Berlin-Friedrichshain, Germany; 12 Department of Trauma, Hand and Reconstructive Surgery, Rostock University Medical Center, 18057 Rostock, Germany; 13 Departement of Orthopedics and Tramatology, Lausanne University Hospital, Rue du Bugnon 46, 1011 Lausanne, Switzerland; 14 Department for Trauma and Orthopedic Surgery, Center for Musculoskeletal Tumor Medicine, Vivantes Hospital Spandau, 13585 Berlin, Germany; 15 Department of Trauma Surgery, Orthopaedics and Sports Traumatology, BG Klinikum Hamburg, Bergedorfer Strasse 10, 21033 Hamburg, Germany; 16 Faculty of Medicine, Department of Orthopedic and Trauma Surgery, Friedrich-Alexander-Universität Erlangen-Nürnberg, Erlangen, Germany; 17 Traumatology and Plastic Surgery, University Centre for Orthopaedics, University Hospital Carl Gustav Carus, University of Technology Dresden, Dresden, Germany; 18 Center for Orthopedics and Trauma Surgery, Klinikum Ingolstadt GmbH, Ingolstadt, Germany; 19 Department of Orthopedics and Trauma Surgery, Medical Center, Faculty of Medicine, University of Freiburg, 79106 Freiburg, Germany; 20 Department of Trauma and Reconstructive Surgery, RWTH Aachen University, 52074 Aachen, Germany; 21 Department of Trauma Surgery and Orthopaedics, RoMed Klinikum Rosenheim, Rosenheim, Germany; 22 Orthopaedics and Spinal Therapy, Catholic Hospital “St. Johann Nepomuk” Clinic for Trauma Surgery, Haarbergstraße 72, 99097 Erfurt, Germany

**Keywords:** Weekend effect, Pelvic trauma, Acetabular fracture, Pelvic ring fracture, Treatment quality, Injury pattern, Mortality, Complication, Health care, Fracture repair

## Abstract

Treatment of pelvic fractures requires extensive human and material resources. The weekend is characterized by a reduced availability of these resources. In addition, weekend leisure activities lead to different injury patterns. The ‘weekend effect’, which describes these conditions, is controversially discussed in medicine. However, there is still a paucity of data, especially in traumatology and particularly in relation to pelvic injuries. The aim of this work is to assess the weekend effect on demographics, injury patterns and outcome in relation to the day of the accident. Demographic, clinical and operative parameters from the data of the German Pelvic Trauma Registry were retrospectively evaluated (n = 16,359). Differences between weekend and weekday accidents were statistically evaluated. Weekend accidents affect younger, more severely injured and less often female patients with fewer displaced fractures and a lower proportion of acetabular fractures. This results in less frequent operative treatment, but more emergency and early definitive surgery. In contrast to the numerous and significant differences in baseline conditions, the outcome in terms of quality of surgical treatment, morbidity and mortality showed only marginal and non-significant differences between weekend and weekday accidents. Weekend accidents differ from weekday accidents in their initial conditions. This does not lead to more frequent—yet more emergency and more early definitive surgeries. However, there are no differences in the quality of care or outcome according to the day of the accident.

## Introduction

Pelvic fractures are frequently serious, occasionally life-threatening, and are often associated with a complex pattern of injury that places significant demands on trauma surgical care. Even in hospitals with maximum care, the required high level of trauma surgical competence and resource capacity, not only on the medical side but also on the nursing side and, for example, on the radiological side, may not be as readily available at weekends as it is on weekdays.

The “weekend effect”^1^, also referred to in economic literature, describes a phenomenon whereby inconsistent results are observed due to the influence of varying conditions on different days. The “weekend effect” has also been the focus of medical research for some time, including trauma surgery research^2–5^. The impact of reduced human and technical resources on clinical outcomes has been extensively studied and documented in the context of various diseases and injuries. This topic has been extensively reviewed in the literature^3,4,6^.

Examples from internal medicine in which the weekend effect has already been shown are mortality in pulmonary embolism^7^ and in cardiac catheterization for myocardial infarction^8^. In surgery, a day-dependent effect was found in surgical procedures in elective colon and pancreatic head cancer surgery^9^

Conversely, a substantial body of research in both traumatology^10^ and internal medicine has failed to substantiate the "weekend effect," or has demonstrated its non-persistence after adjusting for the severity of disease or injury^11^. For instance, Giannoudis et al.^2^ discovered that the patient population admitted on weekends was significantly younger and more severely injured than that admitted on weekdays. However, after adjusting for these differences, no increased mortality was observed on weekends. Similarly, Metcalfe et al. demonstrated that the “weekend effect” no longer persisted after correction for potential confounding variables^5^. Carr et al. were similarly unable to demonstrate the “weekend effect” in traumatology, attributing this to the high level of around-the-clock care that had already been established in acute traumatology^12^.

Görz et al. consider established uniform treatment protocols to be a cause for the non-occurrence of weekend effects^13^. On the other hand, the time until the provision of a special therapy that is not maintained around the clock every day was found to be a cause for the occurrence^14,15^.

Görz et al. ascribed the absence of a discernible “weekend effect” in cerebral aneurysm clipping to the observance of established, uniform treatment protocols^13^. In patients with upper gastrointestinal bleeding, admission during the weekend has been demonstrated to be associated with a 15% increase in mortality among those with non-variceal bleeding, but not in those with variceal bleeding. One potential explanation for this discrepancy is the time required for endoscopy, which was significantly longer at the weekend^14,15^. In consideration of the aforementioned studies, as well as the observations documented by Chen et al. in their 2019 review, it becomes evident that the presence, impact, and scope of the “weekend effect” in medicine exhibit considerable heterogeneity^16^. However, it is frequently unclear to what extent the results can be attributed to overlapping effects in terms of the patient population, injury mechanism, injury pattern or treatment strategies^11^. For instance, Chen et al. indicate that fewer patients who are more seriously ill or injured are admitted during weekends^16^, and that, in contrast to the findings of Görz et al., treatment pathways are often not standardized^13^ and differ between weekends and weekdays. Nevertheless, the role of the “weekend effect” in the epidemiology and care of pelvic fractures remains entirely unclear. Accordingly, this study aims to elucidate the discrepancies between weekends and weekdays, as well as the impact of the day of the accident on morbidity, mortality, and quality of care. This information could be utilized effectively in the context of preventive campaigns. In order to achieve this, it is essential to consider the potential differences between weekend and weekday presentations in terms of patient-specific factors and injury patterns. This will enable the preparation of effective strategies to meet the anticipated demands on the day in question. This retrospective evaluation of 16,359 patients enrolled in the German Pelvic Registry (GPR) from 2003 to 2017 aims to address these questions and develop action strategies for care planning. The study will demonstrate the extent to which the day of the accident influences the outcome of pelvic fractures.

## Patients and methods

In this analysis, data from the German Pelvic Registry (GPR) were subjected to examination. Data on patients with pelvic fractures were collected at 39 German trauma centers between 2003 and 2017. The participating hospitals collected anonymized data on all patients who were treated for a pelvic fracture in the specified period using standardized questionnaires. Informed consent was obtained from each patient. The data collection process was conducted in accordance with the standards approved by the Ethics Committee of the Medical Association of Saarland (No. 29/14). The data analysis was conducted in accordance with the corresponding ethics vote of the local ethics committee of the Eberhard-Karls University in Tübingen, Germany (114/2021BO2).

### Patient selection and models

In this retrospective study, all 16,359 registered cases were included and initially assessed with descriptive statistics, with a particular focus on identifying differences between cases occurring on weekends and on weekdays. To ascertain the baseline conditions, a comparison was made between the variables of age, sex, injury severity and fracture type between accidents occurring on weekends and those occurring on weekdays.

Subsequently, the differences in treatment and clinical course between all weekend and weekday accidents were compared. This involved a comparison of the type of treatment, including the rate of operative and non-operative treatment, and the overall clinical outcome with length of hospital stay (LOHS), morbidity and mortality (Table 2).

In addition, the outcomes of surgically treated weekday and weekend accidents were compared. The duration of surgery, blood loss and fracture displacement were evaluated, as well as the postoperative outcome, including the LOHS, morbidity and mortality (Table 3).

### Definition of the variables

- ISS: Stands for the injury severity score. The injury severity was assessed according to the ISS-system^17^.
- Polytrauma: The GPR database distinguishes polytrauma patients (multiple injuries including the pelvis with an ISS ≥ 16) from patients with isolated pelvic fractures or multiple trauma patients (multiple injuries including the pelvis with an ISS < 16).
- Stable/unstable fracture: The classification of pelvic ring fractures is according to Tile, who respects the degree of instability of the pelvic girdle. In daily practice, the pelvic ring injury is divided into a stable and unstable type whereby type B and C fractures are not considered stable.^18,19^.
- Emergency stabilization: The term was defined in the data set as an initial stabilization procedure performed in an emergency situation. It is employed to stabilize vital functions, particularly those pertaining to circulation, or to reposition or stabilize displaced or displacement-prone fractures in an emergency setting. These include, in particular, the control of bleeding via ligation, embolization, or packing, as well as the application of an external fixator or pelvic clamp.
- Definitive stabilization: Was defined as surgical treatment beyond that which is required in an emergency situation. This may include the use of plates or screw osteosynthesis.
- Early definitive stabilization: Was defined as a definitive treatment that took place within the first five days following the accident.
- LOHS: Means “Length of hospital stay” and quantifies the number of inpatient treatment days from emergency admission to discharge home or follow-up treatment, e.g. rehabilitation.
- Morbidity: Was defined as the occurrence of complications, in particular bleeding, thrombosis, infections, wound healing disorders, general pulmonary or circulatory problems and secondary displacement as well as material failure.
- Fracture displacement: In the case of acetabular fractures, surgical intervention is not advised when the remaining intact roof-arc angle exceeds 40°, the fracture step is less than 1 mm, the fracture gap is smaller than 3 mm, or ideally less than 1 mm, and the comminution zone does not exceed 50%, or is ideally not present at all^20–26^. Accordingly, the cut-off values were set within these variables^27–29^. In order to quantify the displacement of pelvic ring fractures, the maximum displacement in millimeters was evaluated.

### Statistical analysis

For categorical variables, differences were evaluated using the chi-squared test. For continuous variables, the t-test was used.

The significance level was set at 5% (p = 0.05). All analyses were performed using RStudio, version 1.2.5001^30^.

## Results

### Baseline characteristics (Table 1)

**Table 1:** Basic data and fracture distribution.

	Accidentday		p-value
	Weekday	Weekend	
Total number (n)	75.9% (12,414/16,359)	24.1% (3945/16,359)	–
Age (years)	62.20 (± 23.10)	58.79 (± 24.65)	p < 0.001
Gender			
Male (n)	46.0% (5705/12,414)	48.3% (1906/3945)	p = 0.010
Female (n)	54.0% (6709/12,414)	51.7% (2039/3945)	p = 0.010
Injury severity			
ISS	14.14 (± 11.30)	15.13 (± 12.25)	p < 0.001
Polytrauma (n)	21.9% (2713/12,414)	26.4% (1041/3945)	p < 0.001
Type of fracture			
Pelvic ring fracture (n)	75.7% (9403/12,414)	78.2% (3085/3945)	p = 0.002
Stable PR fracture (tile type A) (n)	36.6% (3442/9403)	38.6% (1190/3085)	p = 0.052
Unstable PR fracture (tile type B/C) (n)	63.4% (5961/9403)	61.4% (1895/3085)	p = 0.052
Acetabular fracture (n)	28.1% (3492/12,414)	26.7% (1055/3945)	p = 0.094
Combined pelvic ring + acetabular fracture (n)	6.9% (858/12,414)	7.8% (306/3945)	p = 0.078

*ISS* Injury severity score, *PR* Pelvic ring.

**Fig. 1:**
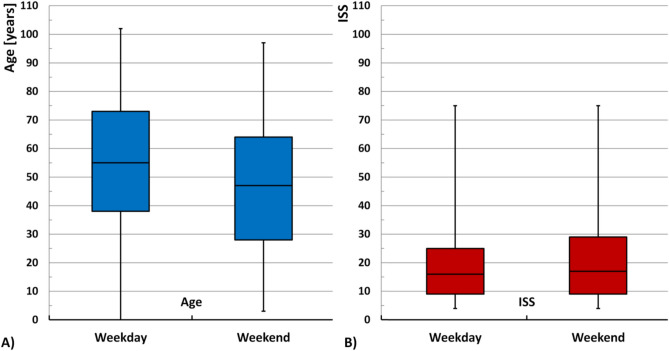
Comparison of (**A**) age [years] and (**B**) ISS (Injury severity score) between weekdays and weekends.

The mean age of patients sustaining a pelvic fracture on weekdays is higher than on weekends (62.20 (± 23.10) vs. 58.79 (± 24.65) years; p < 0.001). In contrast, the severity of injury for pelvic fracture accidents was higher on weekends than on weekdays, as evidenced by both the Injury Severity Score (ISS) (mean 14.14 (± 11.30) vs. 15.13 (± 12.25); p < 0.001) (see also Fig. 1) and the proportion of polytrauma patients (21.9% vs. 26.4%; p < 0.001). The proportion of female patients is higher for accidents occurring on weekdays than on weekends (54% vs. 51.7%; p = 0.010). The proportion of acetabular fractures among pelvic injuries occurring on weekdays is higher than on weekends, although this difference is not statistically significant (28.1% vs. 26.7%; p = 0.094). The proportion of unstable pelvic ring fractures (tile type B/C) is comparable between weekday and weekend accidents (63.4% vs. 61.4%; p = 0.052). This discrepancy does not attain statistical significance (Table 3).

### Clinical course (Table 2)

A higher proportion of patients injured at the weekend are treated conservatively (60.7% vs. 57.5%; p < 0.001), but also undergo emergency stabilization more frequently (44.2% vs. 35.6%; p < 0.001). Furthermore, where surgery was performed, the proportion of patients who underwent early definitive treatment was higher (65.2% vs. 46.3%; p < 0.001). No differences were observed in clinical outcomes, including length of hospital stay, morbidity, and mortality.

**Table 2:** Clinical course.

	Accidentday		p-value
	Weekday	Weekend	
Type of treatment			
Non operative (n)	57.5% (7134/12,414)	60.7% (2395/3945	p < 0.001
Operative (n)	42.5% (5280/12,414)	39.3% (1550/3945)	p < 0.001
Emergency stabilization (n)	35.6% 1881/5280	44.2% 685/1550	p < 0.001
Definitive stabilization (n)	90.9% (4797/5280)	89.6%1389/1550	p = 0.156
–Early definitive therapy (≤ 5 days) (n)	46.3% (2221/4797	65.2% (906/1389	p < 0.001
Clinical course (overall)			
LOHS (days)	17.93 (± 18.82)	18.00 (± 19.92)	p = 0.834
Morbidity (n)	14.4% (1782/12,414)	14.1% (557/3945)	p = 0.732
Mortality (n)	4.0% (493/12,414)	4.3% (169/3945)	p = 0.411

*LOHS* Length of hospital stay.

### Surgical outcome (Table 3)

No difference was observed in the operative time and intraoperative blood loss between accidents occurring on weekdays and those occurring on weekends. In the case of acetabular fractures, the proportion of cases exhibiting a preoperative step of > 2 mm (52.3% vs 48.1%; p = 0.019) and gap > 1 mm (76.5% vs. 71.8%; p = 0.002) is higher on weekdays than on weekends. However, no difference was observed postoperatively. There is no significant difference in fracture displacement in pelvic ring fractures between weekday and weekend accidents.

**Table 3:** Surgical outcome.

	Accidentday		p-value
	Weekday	Weekend	
Operative total number (n)	42.5% (5280/12,414)	39.3% (1550/3945)	p < 0.001
Duration of surgery (min)	183.24 (± 93.97)	186.95 (± 98.85)	p = 0.799
Blood loss (ml)	628.46 (± 546.29)	597.17 (± 493.12)	p = 0.655
Fracture displacement			
Step PreOP acetabulum > 2 mm (n)	52.3% (1774/3390)	48.1% (495/1029)	p = 0.019
Step PostOP acetabulum > 2 mm (n)	14.5% (307/2119)	14.4% (86/597)	p = 1.000
Gap PreOP acetabulum > 1 mm (n)	76.5% (2594/3390)	71.8% (239/1029)	p = 0.002
Gap PostOP acetabulum > 1 mm (n)	43.1% (913/2119)	41.9% (250/597)	p = 0.631
Step PreOP pelvic ring (mm)	8.91 (± 14.79)	9.68 (± 20.31)	p = 0.132
Step PostOP pelvic ring (mm)	6.03 (± 6.10)	5.82 (± 5.93)	p = 0.360
Clinical course (OP)			
LOHS (days)	24.7% (± 19.74)	25.6% (± 21.09)	p = 0.493
Morbidity (n)	5.1% (207/5280)	5.0% (77/1550)	p = 0.081
Mortality (n)	24.9% (1316/5280)	25.2% (390/1550)	p = 0.876

*LOHS* Length of hospital stay.

## Discussion

### Baseline characteristics

A comparison of the initial severity of injury according to the day of the accident reveals that both a higher Injury Severity Score (ISS) and a higher proportion of polytrauma patients are present at weekends. It can be concluded that patients who are involved in accidents at the weekend are more severely injured.

Other important factors are age and sex. In the case of pelvic fractures, weekend accidents tend to affect a younger, more often male group of patients (see also Fig. 1).

Prior research has demonstrated that the severity of injury is greater in males than in females, and this is also the case for younger patients compared to older ones^27^. It may therefore be posited that the disparate risks associated with various leisure and occupational activities between old and young, but also between the sexes, may help to elucidate the observed phenomena.

However, the characteristics of the pelvic fracture itself also have an important bearing on the treatment and course of pelvic fractures. The incidence of acetabular involvement does not differ significantly between accidents occurring on weekends and those occurring on weekdays. Furthermore, there is a notable discrepancy in the severity of acetabular fractures between weekend and weekday accidents. Acetabular fractures occurring on weekends have a significantly lower proportion of fracture steps > 2 mm and fracture gaps > 1 mm. Therefore, the proportion of more displaced acetabular fractures is greater on weekdays. There is no significant difference in pelvic ring displacement between fractures sustained on weekends and those sustained on weekdays.

### Treatment

It is also important to consider the extent to which there are differences in treatment strategy between weekends and weekdays. The proportion of pelvic fractures treated surgically is significantly lower when the accident occurs on a weekend than on a weekday. However, a higher proportion of weekend fractures undergo emergency stabilization and primary care within 5 days of the accident than weekday fractures. This can be attributed, at least in part, to the differing injury patterns and patient demographics observed on weekends. Patients presenting on weekends tend to be younger, more often male, and more severely injured (ISS and Polytrauma), with less complex fracture patterns (fracture displacement). This may result in a different prioritization and treatment strategy than that employed for fractures occurring on weekdays, where the pelvic fracture is given greater weighting in relation to the overall injury pattern. To illustrate, a younger, healthier patient with a more severe overall injury is more often treated more aggressively and will therefore more frequently receive surgical treatment. Furthermore, emergency surgical intervention is frequently required for other reasons in conjunction with polytrauma. An illustrative example is a circulatory problem (C problem according to the ATLS definition^31^) with abdominal bleeding. In such cases, surgical treatment of a pelvic fracture could be performed more frequently where surgical treatment is possible but not absolutely mendatory. Nevertheless, despite these discrepancies, there are no differences in morbidity and mortality. This result is at odds with the findings of Schwartz et al.^32^, who posed the following question: “Are we currently delivering two standards of care for pelvic trauma, depending on the day and time of admission?” Following an evaluation of the time to angioembolization for pelvic fractures in patients admitted at night and on weekends versus those admitted at daytime during the week, it was found that the former group experienced a significant increase in time to angioembolization, which was associated with an almost 100% increase in mortality. As previously stated, this discrepancy in outcomes could not be replicated in the context of our data set as a whole. This discrepancy may be attributed to the fact that the disparities in angioembolization accessibility may not manifest with the same degree of clarity as observed in the single-center study by Schwartz et al.^32^. Either because embolization is not available at all in some centers or is available in the same quality in others regardless of the day of the week.

### Outcome

The pivotal issue in examining the weekend effect is to ascertain whether there are discernible differences in outcomes between weekends and weekdays.

The proportion of patients who die shows no statistically significant difference between those with pelvic fractures who were injured on weekends and on weekdays. This is also the case with regard to the proportion of patients who experience a complication.

In addition to the difference in mortality and morbidity between weekends and weekdays, the quality of the treatment of the pelvic fracture itself also plays an important role. The postoperative fracture step in pelvic ring fractures demonstrates no statistically significant difference between weekends and weekdays when the day of the accident is taken into account. Furthermore, there is no significant difference between the postoperative fracture step and fracture gap for acetabular fractures in relation to the occurrence of pelvic fractures on weekends and weekdays.

A frequently employed criterion in quality assessment is operating time. In addition to the complexity of the injury being treated, it is directly related to the skill and experience of the surgeon. There was no significant difference in operating time between weekend and weekday pelvic fractures. The same is true for LOHS (length of hospital stay). Despite the higher severity of weekend accidents, there were no differences between weekend and weekday accidents in this regard.

### Study limitations and context

The present study examines the influence of weekdays and weekends on pelvic fractures, firstly in relation to injury patterns and demographics, and secondly in relation to quality of care and outcome.

The decision was taken not to exclude any particular groups of patients a priori, such as minors or those with critical injuries, in order to ascertain how pelvic fractures differ on weekends and working days. It was felt that excluding a group, for example by setting artificially limits on age or severity of injury, would risk distorting these differences, which in turn could distort the evaluation of treatment strategy and quality.

The existence and extent of a potential weekend effect in traumatology remains a topic of contention, with conflicting views on the factors contributing to this phenomenon^2,10,33,34^. In particular, data on the weekend effect in the context of pelvic fractures are scarce. The findings of this study indicate that there are notable differences in a number of aspects pertaining to the occurrence of accidents at the weekend compared to those that occur on weekdays. Prior studies have similarly indicated that weekend patients are more often male and younger^33,34^, and a higher severity of injury is also described by Giannoudis et al.^2^. It is not possible to ascertain the reasons for this from the data available. However, it seems reasonable to hypothesise that more risky leisure activities among young men at weekends, such as motorcycling or home maintenance, may be a contributing factor^35,36^.

These findings contrast with the conclusions drawn by Sheikh et al. from their results published in 2017^37^. In this study of the weekend effect in hip fractures, no significant weekend effect was identified, and mortality was found to be predominantly influenced by patient comorbidities and delay to surgery. However, it should be noted that hip surgery is much more common than pelvic fractures, and all those involved in the treatment are likely to have a higher level of experience and training^37^. As the study is retrospective in nature and the GPR only provides data on inpatients without further follow-up, it is important to consider the limitations of this study when interpreting the identified factors. For instance, the study design does not permit the exclusion of selection bias, and the absence of follow-up precludes a definitive statement regarding long-term outcomes. Nevertheless, this study provides a comprehensive overview of the initial inpatient course and represents an important initial step in evaluating the weekend effect in pelvic surgery. To address the remaining questions and new issues raised by this study, further research is required. Ideally, this should be in the form of prospective studies with follow-up.

### Importance of this study

This study demonstrates the differences in characteristics and demographics between pelvic fractures occurring on weekdays and weekends. Furthermore, it illustrates the differences in treatment strategy and quality of care, taking into account the found different baseline conditions. Despite these differences, this does not lead to differences in outcome. This is pleasing but cannot be explained from the available data. Either the different treatment strategies are sufficient to compensate for the differences in baseline conditions or this is due to differences in resilience between the groups. This prompts the question of whether the optimal treatment is being provided for each population, irrespective of the same outcome being found here. The findings of this study therefore provide a foundation for further research in this area.

Knowledge of the initial conditions and the differences depending on the day of accident can help to optimize the material and personnel resources held available. This is particularly interesting given the increasing economic pressure that hospitals are facing. The found results thus can be used for optimization of treatment strategies and algorithms. An established treatment algorithm improves the quality of care^38^ and clear structures allow for optimization of collaboration and resource availability, the results of this work promise to improve planning in this regard. However, the ultimate decision regarding the treatment strategy remains the responsibility of the treating physician. This decision should be made with a high degree of professional expertise, taking into account a range of factors, including those related to the patient, the fracture, the surgeon, the hospital, and the resources available.

It is also noteworthy that the presented results are highly conducive to targeted prevention strategies, particularly with regard to leisure activities on weekends and occupational health and safety measures on weekdays.

## Conclusion

It can be posited that the weekend effect in pelvic fractures represents a discrepancy in baseline conditions. Younger, more often male, more severely injured patients with less complex fracture patterns were observed in weekend accidents. This results in a higher frequency of operative treatment in accidents occurring on weekdays, but a higher frequency of emergency and early definitive surgery in accidents occurring on weekends. Notwithstanding these discrepancies, this does not result in a more pronounced disparity in the quality of surgical treatment, morbidity, or mortality outcomes.

## Data Availability

The datasets analysed during the current study are not publicly available because they come from the Working Group on Pelvic Fractures of the German Trauma Society and cannot be made publicly available without their consent. But they are available from the corresponding author on reasonable request.
